# Significant decreased CXCR3^+^ phenotype Tfh1 cells in three Good’s syndrome patients

**DOI:** 10.3389/fimmu.2026.1816407

**Published:** 2026-04-23

**Authors:** Yanxia Chen, Junwu Zhang, Jinyao Ni, Jing He, Jinlin Liu

**Affiliations:** 1 Department of Rheumatology, South China Hospital, Medical School, Shenzhen University, Shenzhen, Guangdong, China; 2 Department of Clinical Laboratory, Wenzhou TCM Hospital of Zhejiang Chinese Medical University, Wenzhou, China; 3 Department of Pathology, The First Affiliated Hospital of Wenzhou Medical University, Wenzhou, China; 4 Department of Clinical Laboratory, Zhejiang Provincial People’s Hospital, Hangzhou Medical College, Hangzhou, China; 5 Department of Clinical Laboratory, South China Hospital, Medical School, Shenzhen University, Shenzhen, Guangdong, China

**Keywords:** B cells, CXCR3+Tfh1 cells, Good’s syndrome, hypogammaglobulinemia, immunology

## Abstract

**Background:**

Good’s syndrome (GS) is a rare acquired immunodeficiency defined by the co-occurrence of thymoma and hypogammaglobulinemia. Follicular helper T (Tfh) cells, a specialized CD4+ T cell subset, play a critical role in supporting B-cell antibody production. The status and characteristics of Tfh cells in GS, however, remain largely unexplored.

**Methods:**

This study utilized flow cytometry to analyze CD4+CXCR5+ T cells, along with Tfh1 (CXCR3+CCR6−), Tfh2 (CXCR3−CCR6−), and Tfh17 (CXCR3−CCR6+) subsets, in three GS patients compared to twenty healthy controls (HCs). Serum levels of Th1/Th2/Th17 cytokines were also assessed.

**Results:**

The cohort included a 45-year-old female and a 70-year-old female, both with type AB thymoma, and a 47-year-old male with type A thymoma. All patients exhibited markedly reduced serum IgG levels (0.87 g/L, 1.44 g/L, 3.0 g/L) and a profound reduction or absence of peripheral B cells (0%, 0%, 3.7%). The proportions of CD4+CXCR5+ T cells and total Tfh cells in two patients (Cases 1 & 2) were comparable to HCs, while a significant increase was noted in the third patient (Case 3). A key finding was a consistent and drastic reduction in CXCR3+CCR6− Tfh1 cells across all patients, whereas Tfh2 cell frequencies were significantly elevated. Tfh17 cell percentages and Th1/Th2/Th17 cytokine profiles showed no significant alterations.

**Conclusion:**

Our findings reveal, for the first time, a pronounced decrease in circulating CXCR3+ Tfh1 cells in GS patients, suggesting a potential role for this specific immune imbalance in the disease’s immunopathogenesis.

## Introduction

Good’s syndrome (GS) is an acquired immunodeficiency disorder characterized by the triad of thymoma, hypogammaglobulinemia, and recurrent severe infections, including opportunistic viral and fungal pathogens (1). A hallmark of GS, which distinguishes it from common variable immunodeficiency (CVID), is the near-total absence of peripheral B cells, often accompanied by CD4+ T cell lymphopenia (1). Despite its clinical recognition, the underlying immunopathological mechanisms driving GS remain poorly elucidated, and evidence-based treatment protocols are lacking. Consequently, management relies heavily on early diagnosis and intravenous immunoglobulin replacement to mitigate complications.

Follicular helper T (Tfh) cells, a subset of CD4+ T cells defined by expression of CXCR5, BCL-6, and ICOS, are essential for germinal center formation and B-cell maturation into antibody-producing plasma cells (2, 3). Circulating Tfh (cTfh) cells, which mirror their germinal center counterparts, provide an accessible window for studying humoral immunity (4). The functional heterogeneity of cTfh cells is reflected in distinct subsets—Tfh1 (CXCR3+CCR6−), Tfh2 (CXCR3−CCR6−), and Tfh17 (CXCR3−CCR6+)—with Tfh2 and Tfh17 being particularly effective at supporting B-cell antibody production (5). Aberrations in Tfh cell number or function are implicated in various immunodeficiencies. For instance, reduced cTfh frequencies are observed in CD40L and ICOS deficiencies (6), while a Th2/Th17-polarized Tfh profile is seen in Wiskott-Aldrich syndrome (7).STAT3 mutations in hyper-IgE syndrome also lead to diminished Tfh-like cells (8). Therefore, Tfh cells are pivotal for initiating the germinal center reaction, a process essential for effective humoral immunity (3). Consequently, deficiencies in Tfh cell number or function can disrupt the formation of B-cell memory, ultimately leading to hypogammaglobulinemia (3). Given the critical role of Tfh cells in B-cell help and the profound B-cell deficiency in GS, a key unanswered question is how the Tfh compartment adapts in this setting.

Based on this established role, we hypothesized that Tfh cell populations would be diminished or altered in patients with GS. To address this, we conducted a detailed phenotypic analysis of Tfh cells in three GS patients with severe B-cell deficiency. Our study aimed to characterize the distribution of Tfh1, Tfh2, and Tfh17 subsets to shed light on a potential immunological mechanism contributing to GS pathogenesis.

## Materials and methods

### Patient

This study enrolled three patients diagnosed with Good’s syndrome (GS). Peripheral blood samples were collected during routine clinical assessments. The remaining blood after testing was utilized for research purposes in accordance with the ethical principles of the Declaration of Helsinki. Twenty age-matched healthy donors without history of autoimmune disorders, malignancies, or active infections were recruited as healthy controls (HCs). Case 1 and the corresponding HCs were sourced from Zhejiang Provincial People’s Hospital. Cases 2 and 3, along with their matched HCs, were from The First Affiliated Hospital of Wenzhou Medical University.

### Flow cytometry

Peripheral blood mononuclear cells (PBMCs) were isolated from approximately 2 ml of whole blood using standard Ficoll-Paque density gradient centrifugation. For immunophenotyping, PBMCs were stained with a panel of fluorescently conjugated surface antibodies for 20 minutes at room temperature in the dark. The antibodies used were as follows (all from BD Biosciences): BV510-anti-CD4 (Cat: 562970), APC-R700-anti-CD25 (Cat: 565106), Alexa Fluor 647-anti-CD127 (Cat: 558598), BV421-anti-CXCR5 (Cat: 562747), PE-anti-CCR6 (Cat: 551773), and PE-Cy7-anti-CXCR3 (Cat: 560831).

Cell populations were defined as follows:

CD4^+^ T cells: CD4^+^;
T follicular regulatory (Tfr) cells: CD4^+^CXCR5^+^CD25^int-hi^CD127^low^;
T follicular helper (Tfh) cells: CD4^+^CXCR5^+^CD25^low^CD127^int-hi^;
Tfh subsets: Based on CXCR3 and CCR6 expression: Tfh1: CXCR3^+^CCR6^−^; Tfh2: CXCR3^−^CCR6^−^; Tfh17: CXCR3^−^CCR6^+^.

The gating strategy was consistent with our previous report (9). Data acquisition was performed on a Navios flow cytometer (Beckman Coulter), and analysis was conducted using Kaluza Analysis software.

### Serum cytokine and immunoglobulin measurement

Venous blood for serum separation was collected in tubes containing a gel separator and clot activator. Samples were centrifuged at 3,000 rpm for 10 minutes to obtain serum. Concentrations of Th1/Th2/Th17 cytokines (IL-2, IL-4, IL-6, IL-10, TNF-α, IFN-γ, and IL-17A) were quantified using the BD™ CBA Human Th1/Th2/Th17 Cytokine Kit (Cat: 560484) according to the manufacturer’s instructions. Serum levels of IgG, IgA, and IgM were determined using an IMMAGE 800 immunochemistry system (Beckman Coulter).

### Clinical immunophenotyping of lymphocytes

Comprehensive lymphocyte subset analysis was performed using whole blood. The following BD Multitest™ reagent kits were employed: CD3FITC/CD16+CD56PE/CD45PerCP/CD19APC (Cat: 340500) and CD3FITC/CD8PE/CD45PerCP/CD4APC (Cat: 340499). Erythrocytes were lysed using ammonium chloride solution, and stained samples were acquired on a BD FACS Canto II flow cytometer. Data were analyzed using BD FACSDiva™ software. CD3+, CD3+CD4+, CD3+CD8+ T cells, CD19+ B cells, and CD3−CD16+CD56+ NK cells were determined. Complete blood counts were obtained using a Sysmex XE-2100™ automated hematology analyzer.

### Statistical analysis

Quantitative data are presented as mean ± standard deviation (SD). Statistical analyses were performed using GraphPad Prism software (version 5.0) or Origin (2017 version).

## Result

### Clinical and immunological characteristics of Good’s syndrome patients

Peripheral blood B cells were absent in case 1 and case 2 GS patient and/or significantly decreased in the case 3 GS patient.

We report the case of a 45-year-old Chinese female. At age 40, she underwent thymectomy for a giant thymoma and the pathological type was AB type thymoma. After that, she had suffered from recurrent pneumonia approximately 5 episodes a year during the previous 5 years. Hypogammaglobulinemia and absent B-cells were reported in several hospitals with the diagnosis of immunodeficiency. Then she was irregularly treated with intravenous immunoglobulins beginning several years after thymectomy. In this admission, initially laboratory results were listed in **Table 1**. Comprehensive clinical evaluation, including history, physical examination, and laboratory tests (**Table 1**), did not reveal evidence of concurrent autoimmune disease. Laboratory results showed panhypogammaglobulinemia (IgG 0.87g/L, IgA 0.11 g/L and IgM<0.04/L) and absent CD19^+^ B cells (0%), but the percentage of CD4^+^T cells was within the reference range. Furthermore, analysis of bronchoalveolar lavage fluid (BALF) from this patient also confirmed the absence of CD19+ B cells (0%).

**Table 1:** Laboratory Results of these 3 GS patients.

Paremeters	Result					Normal range
Complete blood count	Case 1		Case 2		Case 3	
White blood cell count	3.49		4.91		2.34	3.5-9.5×10^9^/L
Neutrophil (%)	69.4		48.7		57.3	40-75%
Lymphocyte (%)	22.6		34.0		36.8	20-50%
Absolute neutrophil	2.4		2.39		1.3	1.8-6.3×10^9^/L
Absolute lymphocyte	0.8		1.67		0.9	1.1-3.2×10^9^/L
Platelet count	356		161		296	125-350×10^9^/L
Hemoglobin	113		152		91	115-150 g/L
Immunoglobulin						
IgG	0.87			1.44	3.0	7.51-15.60 g/L
IgM	<0.04			0.18	0.29	0.82-4.53 g/L
IgA	0.11			0.26	0.50	0.46-3.04 g/L
Antinuclear antibody	Negative			Negative	Negative	Negative
Hepatitis B, C, HIV and symphilis	Negative			Negative	Negative	Negative
Immunophenotype	Peripheral blood	Bronchoalveolar lavage fluid		Peripheral blood	Peripheral blood	
CD19^+^ B cell (%)	0	0		0	3.7	7-23 %
CD4^+^ T cell (%)	34.5	5.1		15.5	30.9	24.5-41.9%
CD8^+^ T cell (%)	58.8	85.3		72.9	57.3	15.6-31.4%
CD3^−^CD16^+^CD56^+^ NK cell (%)	5.5	2.1		8.0	2.5	8-20%

Case 2 was a 47-year-old man presented with recurrent cough, expectoration and received the thymomectomy in 2016, which identified type A thymoma. He was frequently admitted to the outpatient clinic to receive IVIG treatment. Recently, he had stopped IVIG treatment for economic reasons. Laboratory results revealed low IgG (1.44 g/L) and absent CD19^+^B lymphocytes (0%). Also the percentage of CD8^+^T cell was higher while CD4^+^T cell was lower (**Table 1**). Autoimmune diseases and other infection diseases were examined and excluded (**Table 1**).

Additionally, a 70-year-old woman presented with recurrent oral lichen planus, skin abscess, recurrent diarrhea and received the thymomectomy in 2016 with the AB type thyoma was also analyzed (Case 3). The basic lab tests revealed low IgG 3.0g/L, IgM 0.29g/L, IgA 0.5g/L and low CD19^+^B cells (3.7%) (**Table 1**). Also, autoimmune diseases and other infection diseases were examined and excluded (**Table 1**). However, this patient had never used the IVIG to boost the immunity. Also the percentage of CD8^+^T cell was higher while CD4^+^T cell was lower, and the percentage of NK cell was decreased in the peripheral blood (**Table 1**).

### The percentages of CD4^+^CXCR5^+^T and Tfh cells of case 1 and case 2 GS patient were not significantly changed, but significantly increased in case 3 GS patient compare to the HCs

**Figure 1:**
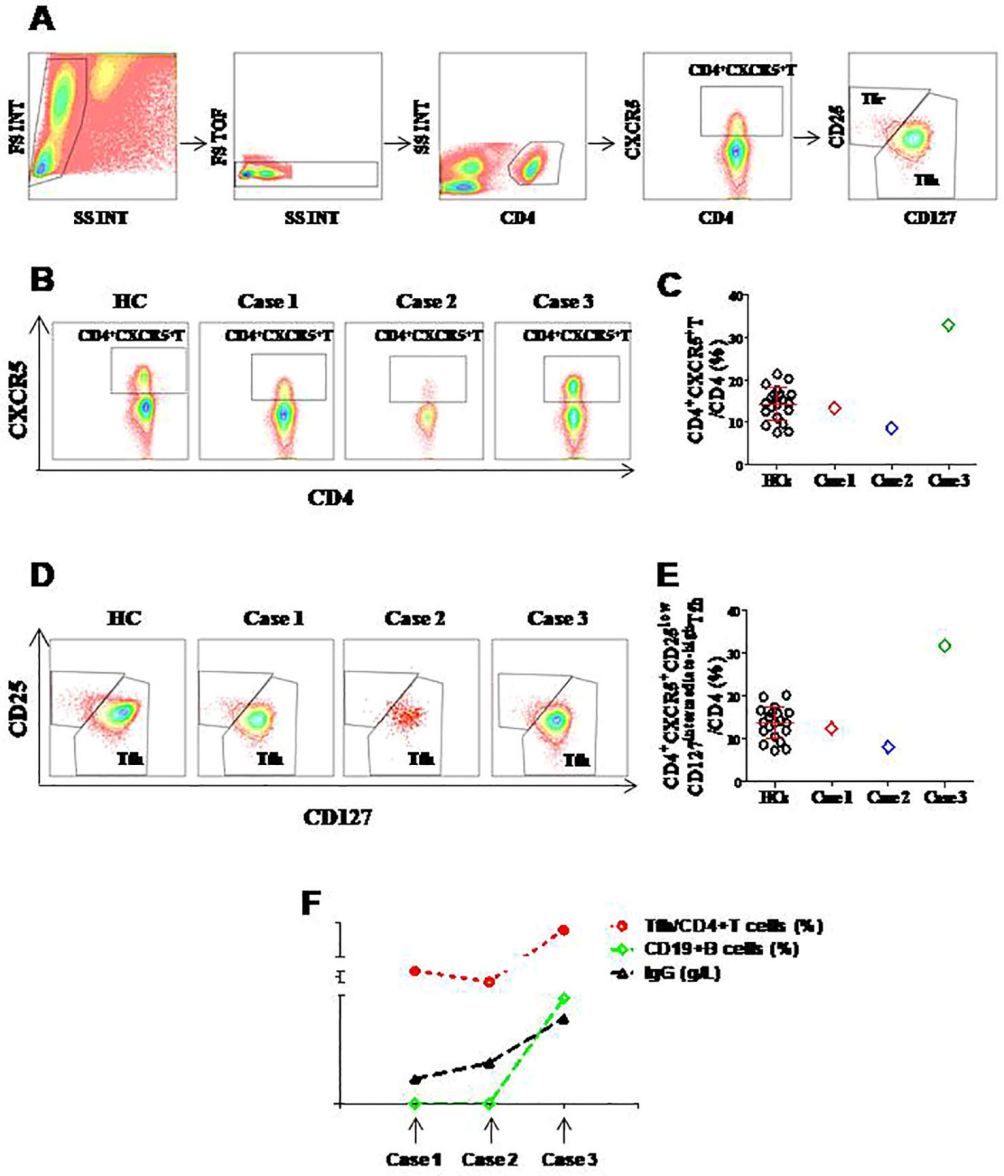
Analysis of circulating CD4+CXCR5+ T and T follicular helper (Tfh) cells in Good’s syndrome (GS) patients compared to healthy controls (HCs). **(A)** Gating strategy for identifying CD4+CXCR5+ T cells and the defined Tfh cell population (CD4+CXCR5+CD25lowCD127int-hi) in peripheral blood. **(B, D)** Representative flow cytometry plots depicting the percentages of CD4+CXCR5+ T cells **(B)** and Tfh cells **(D)** in a healthy control (HC) and the three GS patients (Case 1, Case 2, Case 3). **(C, E)** Quantitative comparison of the percentages of CD4+CXCR5+ T cells **(C)** and Tfh cells **(E)** between the 20 HCs and the 3 GS patients. Data are presented for individual subjects. **(F)** A line chart showing the concurrent measurements in serum IgG levels (g/L), peripheral CD19+ B cell percentages, and Tfh cell frequencies (as a percentage of CD4+ T cells) across the three GS patients.

Given the critical role of Tfh cells in B-cell help, we performed a detailed flow cytometric analysis of CD4+CXCR5+ T cells in peripheral blood samples from our three GS patients and twenty healthy controls (HCs). Our gating strategy involved first identifying the total CD4+CXCR5+ T cell population. From this population, we excluded follicular regulatory T (Tfr) cells (CD4+CXCR5+CD25^int-hi^CD127^low^), which are known to suppress Tfh and B-cell activity, to precisely define the Tfh cell subset for analysis. We then focused on CD4+CXCR5+CD25^low^CD127^int-hi^Tfh cells, a definition consistent with our previous work (9) (**Figure 1A**).

Contrary to our initial hypothesis, the results revealed a patient-specific variation. The percentages of both CD4+CXCR5+ T cells and Tfh cells in Cases 1 and 2 were comparable to those in HCs(**Figures 1B–E**). In striking contrast, Case 3 exhibited a significant increase in these cell populations within the peripheral blood compared to the control group(**Figures 1B–E**). A comparative trend analysis plotting IgG levels, CD19+ B cell percentages, and Tfh cell frequencies across the three patients (**Figure 1F**).

### The percentages of Tfh1 cells were all significantly decreased, but the Tfh2 cells were significantly increased in these 3 GS patients

**Figure 2:**
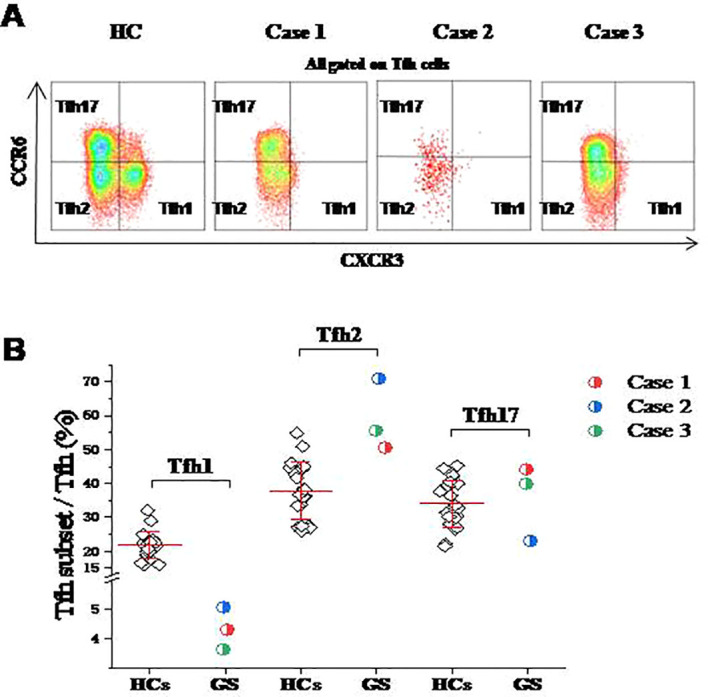
Altered distribution of circulating Tfh cell subsets in Good’s syndrome (GS) patients.**(A)** Representative flow cytometry plots showing the distribution of Tfh1 (CXCR3+CCR6−), Tfh2 (CXCR3−CCR6−), and Tfh17 (CXCR3−CCR6+) subsets within the Tfh cell population from a healthy control (HC) and the three GS patients. **(B)** Comparative analysis of the percentages of Tfh1, Tfh2, and Tfh17 cells among the total Tfh cells between the 20 HCs and the 3 GS patients. Data points represent individual subjects. Data points for individual GS patients (n=3) are shown superimposed on the distribution from HCs (n=20). Given the small patient cohort, data are presented descriptively.

Interestingly, our analysis revealed a striking alteration in the Tfh subset distribution. A profound and consistent reduction in the frequency of CXCR3+CCR6− Tfh1 cells was observed across all three GS patients, with levels barely detectable in some cases (**Figures 2A, B**). In contrast, the proportion of Tfh2 cells was significantly elevated compared to healthy controls. The Tfh17 subset frequency, however, remained largely unchanged, notwithstanding a slight, non-significant increase in Patients 1 and 3 (**Figures 2A, B**). These findings suggest that the observed imbalance within the circulating Tfh compartment—specifically, the severe Tfh1 deficit with a concomitant skew toward the Tfh2 phenotype—may represent a compensatory, albeit ineffective, attempt to stimulate immunoglobulin production in the context of the profound B-cell deficiency characteristic of GS. However, due to the small cohort size (n=3), formal statistical comparison with controls was not performed; data are presented descriptively.

### Th1/Th2/Th17 cytokines in these 3 GS patient were not significantly changed

Furthermore, we quantified the serum levels of key cytokines (IL-2, IL-4, IL-6, IL-10, TNF-α, IFN-γ, and IL-17A) using a multiplex bead assay. In contrast to the cellular findings, the analysis revealed no significant alterations in the concentrations of these Th1/Th2/Th17-associated cytokines in the GS patients compared to reference ranges (**Table 2**).

**Table 2:** Th1/Th2/Th17 cytokine in these 3 GS patients.

Cytokine	Concentration (Case 1)	Concentration (Case 2)	Concentration (Case 3)	Reference value	Unit
IL-2	1.12	0.01	0.95	0.0-4.1	pg/mL
IL-4	1.89	0.01	1.33	0.0-3.2	pg/mL
IL-6	4.41	2.97	7.66	0.0-5.0	pg/mL
IL-10	6.57	0.45	3.45	0.0-5.0	pg/mL
IL-17A	1.44	0.70	1.16	0.0-5.9	pg/mL
TNF-α	1.05	0.81	0.97	0.0-6.0	pg/mL
IFN-γ	7.02	2.06	1.39	0.0-6.0	pg/mL

## Discussion

The link between thymoma and adult-onset hypogammaglobulinemia, first identified by Dr. Good in 1955, defines Good’s syndrome (GS) (10). While over 160 cases have been documented in the literature, the underlying pathogenic mechanisms remain largely enigmatic (11). The characteristic immunological profile of GS includes hypogammaglobulinemia, a profound deficiency or absence of B cells, an altered CD4+/CD8+ T-cell ratio, CD4+ T-cell lymphopenia, and impaired T-cell function (1). It is noteworthy that a comprehensive immunological workup is reported in only a quarter of published cases, likely reflecting the rarity of this condition (11). Several hypotheses have been proposed to explain the thymoma-immunodeficiency link. One suggests that bone marrow stromal cell-derived cytokines may simultaneously affect thymic and B-cell precursors, while another posits that thymoma-derived T cells directly inhibit pre-B-cell development and antibody production (12). However, neither theory has gained broad acceptance (11, 12), highlighting the need for alternative pathophysiological models.

In primary immunodeficiencies, circulating T follicular helper (Tfh) cell frequencies are often severely reduced, underscoring a reliance on B cells for Tfh cell maintenance in humans (13). In contrast, GS represents a secondary immunodeficiency. Our analysis of three GS patients revealed a systemic B-cell deficiency, evident in both peripheral blood and bronchoalveolar lavage fluid. Intriguingly, despite this B-cell void, the total circulating Tfh cell count was largely preserved in two patients (Cases 1 and 2) and even elevated in one (Case 3). This observation aligns with studies showing that B-cell depletion in humans has minimal effects on the lymph node Tfh pool (14), indicating that B cells may not be essential for maintaining the circulating Tfh compartment in this context. A pivotal finding was the consistent and severe reduction in the Tfh1 subset (CXCR3+CCR6−), coupled with a skewing towards Tfh2 cells across all patients. Given that Tfh2 and Tfh17 cells are potent inducers of B-cell antibody production (15), this specific imbalance may represent a compensatory, albeit ineffective, immunological response aimed at rescuing humoral immunity despite a severely limited B-cell repertoire. The body may be attempting to counteract the humoral deficit by promoting Tfh subsets capable of providing maximal B-cell help, even in the face of a severely limited B-cell pool. This model is supported by the elevated total Tfh cells in Case 3, potentially indicating a more vigorous but ultimately insufficient compensatory effort.

This concept of a skewed, potentially compensatory Tfh response stands in contrast to the earlier hypothesis that thymoma-derived T cells universally suppress B-cell function (12). Instead, our data suggest a more complex interplay where the immune system actively, though futilely, attempts to bolster humoral immunity. This interpretation is further nuanced by observations in myasthenia gravis (MG), where thymomas are associated with increased intrathymic Tfh cells that correlate with disease severity (16, 17). This indicates that thymomas can actively shape the Tfh compartment, though the outcome (immunodeficiency in GS vs. autoimmunity in MG) likely depends on the broader immunological context.

Tfh cell differentiation is a complex, multi-step process influenced by various factors (18). It can originate from naïve CD4+ T cells or through the conversion of existing effector cells, such as Th1 cells adopting a Tfh fate (18).For example, in humans, inborn errors that impair B cell development or T-B interactions do reduce the number of circulating CXCR5^+^ T cells; however, B cell depletion with rituximab has little effect on the lymph-node Tfh pool (14). Thus, the impact of acquired B cell deficiency on Tfh maintenance remains unclear. Our findings further support this observation: in our GS patients, the Tfh population is preserved even in the absence of B cells—their primary interaction partners.

It is important to note that cTfh cells, while accessible for study, may not fully recapitulate the biology of Tfh cells within lymphoid tissues. Discordance between peripheral and lymphoid tissue Tfh pools has been documented in other conditions, such as HIV and certain inborn errors of immunity (19–21).Therefore, our findings in peripheral blood warrant cautious interpretation and highlight the need for future studies investigating Tfh cells within lymphoid organs of GS patients, should such tissue become available.

Additionally, the profound deficiency we observed refers specifically to the CXCR3+CCR6- Tfh1 cellular subset. The detectable levels of serum IFN-γ, a canonical Th1 cytokine, in some patients are not necessarily contradictory. Serum cytokines reflect a systemic, mixed immune state and could be produced by other cell types, such as conventional Th1 cells, CD8+ T cells, or NK cells, especially in the context of recurrent infections common in GS. Thus, a phenotypic deficiency in a specific helper T subset does not preclude cytokine production by other components of the immune system. A further limitation is the lack of absolute Tfh cell counts, which precludes a quantitative assessment of these subsets, as absolute counting tubes were not employed. It should also be noted that the relevant patient findings emerged incidentally during a retrospective reanalysis of the data.

In conclusion, our study provides the first evidence of a profound Tfh1 deficiency and a Tfh2-skewed profile in GS. We propose that this specific Tfh imbalance may signify a failed compensatory mechanism aimed at rescuing antibody production.

The main limitation of this study is its small sample size, inherent to the rarity of GS. While our findings reveal a novel and consistent alteration in the Tfh compartment, future investigations with larger cohorts and functional assays are essential to validate these findings and elucidate the precise role of Tfh cells in GS pathogenesis.

## Data Availability

The original contributions presented in the study are included in the article/supplementary material, further inquiries can be directed to the corresponding author/s.
